# Insufficient balloon size limits the interpretation of REBOARREST

**DOI:** 10.1186/s13054-026-06254-9

**Published:** 2026-08-12

**Authors:** Habib Frost, Anette Kristiansen

**Affiliations:** Neurescue ApS, Antonigade 11, Copenhagen, Denmark

Brede et al. should be congratulated for completing the first randomized trial of resuscitative endovascular balloon occlusion of the aorta (REBOA) for out-of-hospital cardiac arrest. However, several features materially limit how the results can be interpreted [1]. 

First, the issue of balloon size deserves explicit attention. Effective aortic balloon occlusion requires adequate balloon apposition to the aortic wall. In the published article, all sites used the REBOA Medical 20 mm balloon, except for one site which used ER-REBOA. The REBOA Medical 20 mm balloon can maximally be inflated to 20 mm diameter. The trial population had a median age of 68 years [1]. In a 70-year-old population, mean descending aortic diameter is 32 mm in men and 28 mm in women, so a 20 mm balloon underinflates the aortic lumen area by 49–61% [2]. 

This is not merely a technical detail because the rationale behind REBOA for cardiac arrest is to maximally redirect the limited CPR-generated blood flow toward the heart and brain, not partial occlusion, and is justified on preclinical studies where complete balloon occlusion of the aorta was performed [3]. 

If the balloon does not provide complete or sufficient occlusion, the intervention being evaluated may not have been provided. Consequently, the study may in part have assessed attempted REBOA deployment rather than effective aortic balloon occlusion [1, 3]. 

Second, REBOARREST may have treated patients too late. The authors acknowledge this. Median time from arrest to balloon inflation was 47 min (IQR 41–54). That interpretation is consistent with ECPR literature, in which outcomes are better when low-flow time is under 40 min [4]. 

Third, the regulatory basis for using the devices in the trial warrants clarification. The study mainly used the REBOA Medical 20 mm balloon catheter [1]. The EU Medical Device Regulation (MDR) became applicable on 26 May 2021, before enrolment began on 7 June 2021 [1, 5]. To our understanding the REBOA Medical 20 mm balloon catheter is not certified with a CE mark under MDR. Although the device had previously obtained a CE mark for hemorrhage control, this indication differs fundamentally from cardiac arrest. Hemorrhage control and cardiac arrest resuscitation involve distinct disease processes, treatment goals, and risk profiles. Therefore, device-specific evidence supporting one indication cannot be assumed to demonstrate its safety or performance for the other.

Could the authors clarify whether use of the devices for cardiac arrest treatment was reviewed and accepted by the competent authorities in all participating countries?

Fourth, the exclusion of enrolled patients after randomization may introduce bias. The authors report that 21 enrolled patients were excluded because deferred consent was not obtained, and that patient health data were removed from the database [1]. In emergency cardiac arrest research, post-randomization removal of data may distort the study population and affect the validity of safety and performance conclusions, particularly when mortality is high and the inability to obtain consent may be related to outcome [6]. 

For these reasons, we suggest that the appropriate conclusion from REBOARREST is that a 20 mm diameter balloon inflated after a median of 47 min from arrest did not improve sustained return of spontaneous circulation compared with advanced life support alone [1]. 

## Data Availability

No datasets were generated or analysed during the current study.

## Abbreviations

ECPR: Extracorporeal cardiopulmonary resuscitation
MDR: Medical device regulation
REBOA: Resuscitative endovascular balloon occlusion of the aorta
